# Antimicrobial Susceptibility of *Streptococcus suis* Isolated from Diseased Pigs in Thailand, 2018–2020

**DOI:** 10.3390/antibiotics11030410

**Published:** 2022-03-18

**Authors:** Kamonwan Lunha, Wiyada Chumpol, Sukuma Samngamnim, Surasak Jiemsup, Pornchalit Assavacheep, Suganya Yongkiettrakul

**Affiliations:** 1 National Center for Genetic Engineering and Biotechnology, National Science and Technology Development Agency, Pathum Thani 12120, Thailand; wiyada.chu@ncr.nstda.or.th (W.C.); surasak@biotec.or.th (S.J.); 2 Department of Veterinary Medicine, Faculty of Veterinary Science, Chulalongkorn University, Bangkok 10330, Thailand; sukuma06@yahoo.co.th (S.S.); pornchalit.a@chula.ac.th (P.A.)

**Keywords:** antimicrobial resistance, AMR, multidrug resistance, MDR, surveillance, zoonosis

## Abstract

*Streptococcus suis* is a porcine and zoonotic pathogen that causes severe systemic infection in humans and pigs. The treatment of *S. suis* infection relies on antibiotics; however, antimicrobial resistance (AMR) is an urgent global problem, pushing research attention on the surveillance of antibiotic-resistant *S. suis* to the fore. This study investigated the antimicrobial susceptibility of 246 *S. suis* strains isolated from diseased pigs in Thailand from 2018–2020. The major sources of *S. suis* strains were lung and brain tissues. PCR-based serotyping demonstrated that the most abundant serotype was serotype 2 or ½, followed by serotypes 29, 8, 9, and 21. To the best of our knowledge, this is the first report describing the distribution of AMR *S. suis* serotype 29 in diseased pigs. The antimicrobial susceptibility test was performed to determine the minimum inhibitory concentrations of 35 antimicrobial agents. The results showed that important antimicrobial agents for human use, amoxicillin/clavulanic acid, daptomycin, ertapenem, meropenem, and vancomycin, were the most effective drugs. However, a slight decrease in the number of *S. suis* strains susceptible to amoxicillin/clavulanic acid and vancomycin raised awareness of the AMR problem in the future. The data indicated a tendency of reduced efficacy of available veterinary medicines, including ampicillin, cefepime, cefotaxime, ceftiofur, ceftriaxone, chloramphenicol, florfenicol, gentamicin, penicillin, and tiamulin, for the treatment of *S. suis* infection, thus emphasizing the importance of the prudent use of antibiotics. The widespread of multidrug-resistant *S. suis* strains was identified in all serotypes and from different time periods and different regions of the country, confirming the emergence of the AMR problem in the diseased pig-isolated *S. suis* population.

## 1. Introduction

*Streptococcus suis* is an important swine pathogen that is responsible for a variety of infections, such as meningitis, septicemia, arthritis, endocarditis, and sudden death, leading to high mortality and considerable economic losses. Moreover, as an emerging zoonotic pathogen, it also has public health implications in humans who come in contact with infected pigs or contaminated pork products [1,2]. Based on the capsular polysaccharides, there are 35 recognized serotypes (serotypes 1–34 and ½) of *S. suis*. However, recent taxonomic studies using DNA-based approaches have reclassified six of those serotypes as *Streptococcus orisratti* (serotypes 32 and 34), *Streptococcus parasuis* (serotypes 20, 22, and 26), and *Streptococcus ruminantium* (serotype 33) [2,3]. Serotype 2 is the most prevalent in pathogenic *S. suis* infection, and the other serotypes, including serotypes 1, 3, 4, 5, 7, 8, 9, 14, 16, 21, 24, and 31, have also been associated with diseases in pigs and humans [2,4].

Because of antigenic variations, no effective vaccines against specific serotypes of *S. suis* are available. Therefore, antimicrobial agents play an important role in treating and controlling *S. suis* infection [2], while an evident overlap between the use of antimicrobials in the livestock sector and human medicine exists [5]. Beta-lactams, tetracyclines, sulfonamides, and macrolides are the most frequently used antimicrobials for the treatment of streptococcal infections in humans and pigs. However, a large body of knowledge has shown the growing trend of resistance in a zoonotic pathogen *S. suis* to commonly used antibiotics, including tetracyclines and macrolides, worldwide [6,7,8].

Despite intensive use of antimicrobials and growing evidence of the emergence of antimicrobial resistance in *S. suis* worldwide, there have been limited investigations on the prevalence and antibiotic resistance of this organism in Thailand. Recently, Yongkiettrakul et al. [9] reported on the antimicrobial resistance (AMR) profiles of *S. suis* strains obtained from asymptomatic pigs, diseased pigs, and human patients in Thailand, highlighting the zoonotic transmission of AMR *S. suis* in Thailand. While beta-lactams, vancomycin, chloramphenicol, and florfenicol remained effective agents with low levels of resistance, high rates of intermediate susceptibility to penicillin were observed, suggesting a progressive trend of rising antibiotic resistance among *S. suis*. Antimicrobial resistance levels can differ among different countries, serotypes, and over a period of time [2,7]. Therefore, it is essential to monitor the antimicrobial susceptibility of *S. suis*, especially in different endemic areas and time periods, for monitoring the emergence of resistant strains and optimizing the available therapeutic options. This study therefore aimed to conduct AMR surveillance to monitor the current emerging AMR situation of *S. suis* isolated from diseased pigs in Thailand.

## 2. Results

### 2.1. Bacterial Sampling

**Figure 1:**
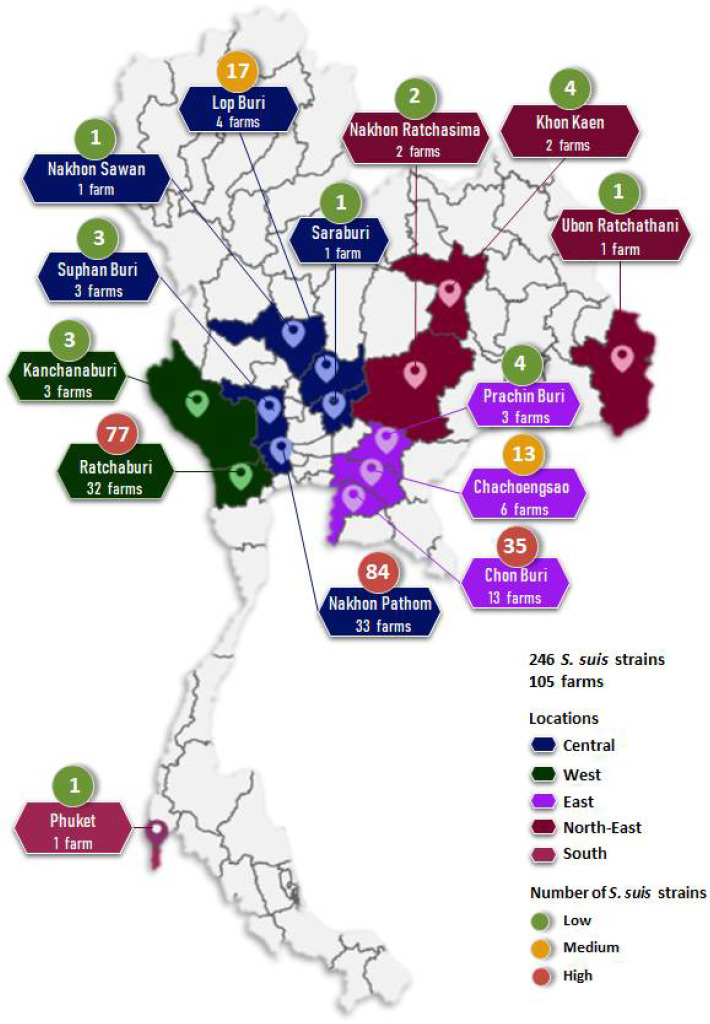
Geographic distribution of *S. suis* strains isolated from diseased pigs from 2018–2020.

**Figure 2:**
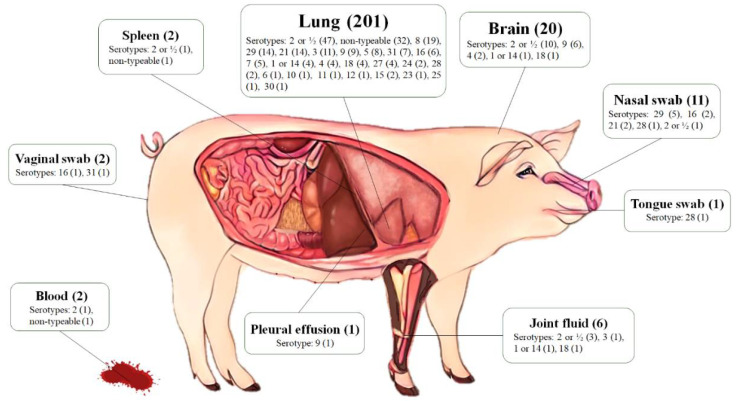
Anatomical origin of 246 *S. suis* strains and their serotype distribution.

A total of 246 *S. suis* strains were recovered from diseased pigs from 2018–2020. The pig specimens were derived from 105 farms localized in 14 provinces across Thailand (Figure 1 and Table S1), including Nakhon Pathom (33 farms, 84 strains), Ratchaburi (32 farms, 77 strains), Chon Buri (13 farms, 35 strains), Chachoengsao (6 farms, 13 strains), Lop Buri (4 farms, 17 strains), Prachin Buri (3 farms, 4 strains), Kanchanaburi (3 farms, 3 strains), Suphan Buri (3 farm, 3 strains), Khon Kaen (2 farms, 4 strains), Nakhon Ratchasima (2 farms, 2 strains), Nakhon Sawan (1 farm, 1 strain), Phuket (1 farm, 1 strain), Saraburi (1 farm, 1 strain), and Ubon Ratchathani (1 farm, 1 strain). *S. suis* strains were isolated from various organs of diseased pigs, including lung (81.7%), brain (8.1%), nasal swab (4.5%), joint fluid (2.4%), blood, spleen, vaginal swab (0.8% each), pleural effusion, and tongue swab (0.4% each) (Figure 2).

### 2.2. Serotyping

PCR-based serotyping revealed that most *S. suis* strains (62.6%) belonged to serotype 2 or ½ (25.6%), followed by serotypes 8 and 29 (7.7% each), 9 and 21 (6.5% each), 3 (4.9%), 16 (3.7%), and other serotypes (23.6%). There were 34 non-typeable strains (13.8%) that could not provide any specific band to all tested multiplex PCR reactions, and no *S. suis* serotypes 13, 17, and 19 were identified from this study (Table 1). It is noteworthy that this PCR-based serotyping protocol did not enable the differentiation of serotype 2 from ½ and 1 from 14.

Lung (*n* = 201, 81.7%), brain (*n* = 20, 8.1%), nasal swab (*n* = 11, 4.5%), joint fluid (*n* = 6, 2.4%), blood (*n* = 2, 0.8%), spleen (*n* = 2, 0.8%), vaginal swab (*n* = 2, 0.8%), pleural effusion (*n* = 1, 0.4%), and tongue swab (*n* = 1, 0.4%) samples were collected.

Regarding the serotypes and anatomical sites of isolation, lung, brain, and nasal swabs comprised 94.3% of the isolation sites. Serotype 2 or ½ was the most prevalent in the isolates recovered from lung (23.4%), followed by serotypes 8 (9.5%), 21, and 29 (7.0% each). The main serotype recovered from the brain was serotype 2 or ½ (50.0%) and 9 (30.0%), while serotype 29 (45.5%) was the most common serotype for nasal swabs. In addition, serotype 2 or ½ was found in almost all types of tissues except tongue, vaginal, and pleural effusion (Figure 2 and Table S2).

### 2.3. Antimicrobial Susceptibility Profiles

The distributions, MIC_50_, MIC_90_, and rate of antimicrobial resistance against the 246 *S. suis* strains isolated from samples collected during 2018 (*n* = 72), 2019 (*n* = 97) and 2020 (*n* = 77) are presented in Table 2. Antimicrobial resistance of zoonotic bacteria is a source of concern, as it may compromise the effective treatment of the infection in humans. In this study, the antimicrobial susceptibility test (AST) was therefore performed to determine minimum inhibitory concentrations (MICs) of 35 antimicrobial agents, covering both veterinary and human medicines. No MIC breakpoints were observed for danofloxacin, sulphadimethoxine, and tulathromycin for *Streptococcus* spp., while concentrations of moxifloxacin and tigecycline used in this study were out of the recommended MIC breakpoint values; therefore, the AST interpretation could be made for 30 of 35 antibiotics according to CLSI veterinary breakpoints, EUCAST, FDA, and previously reported data (Table S3).

The results revealed that *S. suis* strains obtained from diseased pigs remained highly susceptible to meropenem (100%), ertapenem (97.6%), daptomycin (97.2%), vancomycin (97.2%), amoxicillin/clavulanic acid (95.1%), and ceftiofur (85.4%), but considerably resistant to clindamycin (99.6%), tetracycline (99.2%), tilmicosin (98.0%), tylosin tartrate (98.0%), erythromycin (97.2%), azithromycin (96.1%), oxytetracycline (96.3%), and chlortetracycline (95.5%). A high prevalence of *S. suis* resistant to tiamulin (79.3%), cefuroxime (67.9%), trimethoprim/sulfamethoxazole (64.6%), ceftriaxone (62.6%), cefotaxime (59.8%), spectinomycin (55.3%), and enrofloxacin (54.9%) was also detected.

From 2018–2020, intermediate susceptibility to levofloxacin (50.0%), chloramphenicol (29.3%), florfenicol (23.6%), and penicillin (16.7%) were determined (Table 2). In 2020, intermediate susceptibility and resistance to levofloxacin were relatively high (48.1% and 52.0%, respectively), and no susceptible strain to levofloxacin was found (Table 3). In addition, the results demonstrated the high prevalence of penicillin-resistant *S. suis* during 2018–2020 with an increasing penicillin MIC_50_ value from 0.5 µg/mL (in 2018) to 2.0 µg/mL (in 2020) and a constant MIC_90_ value at >8 µg/mL. Among beta-lactam antibiotics, ertapenem and meropenem exhibited the highest activity, with >97.0% of the isolates being susceptible. A high prevalence of antimicrobial susceptibility to amoxicillin/clavulanic acid (93.0–96.9%), vancomycin (95.9–98.6%), and daptomycin (96.0–98.0%) was also determined (Tables S4–S6). Among the 3rd generation cephalosporins, ceftiofur was the most effective drug (80.5–90.3%), whereas a low prevalence of antimicrobial susceptibility to ceftriaxone (33.8–41.2%), and cefotaxime (38.9–42.3%) were reported. However, resistance against ceftiofur emerged (9.7–16.9%). Furthermore, the results demonstrated the presence of strains resistant to the 4th generation cephalosporin, cefepime (26.4–40.3%).

**Figure 3:**
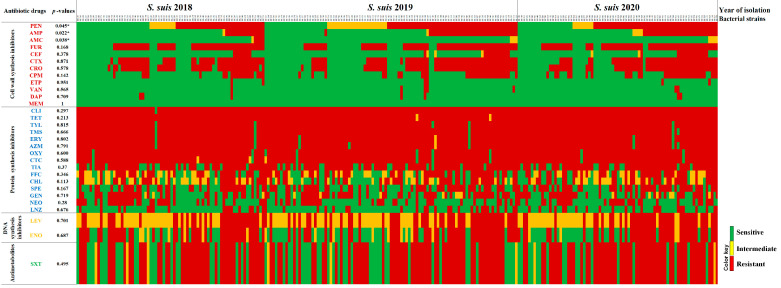
Heat map showing antimicrobial susceptibility profiles of *S. suis* strains. Rows represent antibiotics and columns represent bacterial strains, where green blocks indicate antibiotic susceptibility, yellow blocks indicate intermediate, and red blocks indicate resistance action of the antibiotics. Cell wall synthesis inhibitor antibiotics: AMC, Amoxicillin/Clavulanic acid; AMP, Ampicillin; CEF, Ceftiofur; CPM, Cefepime; CRO, Ceftriaxone; CTX, Cefotaxime; DAP, Daptomycin; ETP, Ertapenem; FUR, Cefuroxime; MEM, Meropenem; PEN, Penicillin; VAN, Vancomycin. Protein synthesis inhibitor antibiotics: AZM, Azithromycin; NEO, Neomycin; CHL, Chloramphenicol; CLI, Clindamycin; CTC, Chlortetracycline; ERY, Erythromycin; FFC, Florfenicol; GEN, Gentamicin; LNZ, Linezolid; NEO, Neomycin; SPE, Spectinomycin; TET, Tetracycline; TMS, Tilmicosin; TYL, Tylosin tartrate; TIA, Tiamulin; OXY, Oxytetracycline. Nucleic acid synthesis inhibitor antibiotics: ENO, Enrofloxacin; LEV, Levofloxacin. Antimetabolite antibiotic: SXT, Trimethoprim/sulfamethoxazole. S, susceptible; I, intermediate; R, resistant. * *p*-value < 0.05.

The AMR profile revealed 208 different antibiograms (patterns of antibiotic resistance), including 152 patterns for 173 *S. suis* strains that exhibited multidrug resistance (MDR) and resistance to at least one agent in three or more antimicrobial categories [10]. None of the *S. suis* isolated strains used in this study was susceptible to all tested antibiotic drugs (Figure 3). The AMR profiles suggested that antibiotics inhibiting cell wall synthesis were the most effective therapeutic drugs (Figure 3). Of 246 *S. suis* strains, 34 strains (13.8%) exhibited antimicrobial susceptibility to all cell wall synthesis inhibitors. There were 71 strains (28.9%) susceptible to all 5 cephalosporins, and a high prevalence of antimicrobial susceptibility to ceftiofur, the 3rd generation drug commonly used in veterinary medicine, was determined.

Regarding antibiotic drugs inhibiting protein synthesis, more than half of the isolates were susceptible to linezolid (73.6%), followed by neomycin (59.8%). For fluoroquinolones, DNA synthesis inhibitor drugs, no *S. suis* strain exhibited antimicrobial susceptibility to levofloxacin, and only 91 strains (37.0%) were susceptible to enrofloxacin. The prevalence of MDR *S. suis* isolated in 2018, 2019, and 2020 was 52 (72.2%), 61 (62.9%), and 60 (77.9%), respectively. MDR *S. suis* strains were found in different regions of the country. The results also revealed a significant association between the isolation period and susceptibility of *S. suis* to amoxicillin/clavulanic acid (*p* = 0.022), ampicillin (*p* = 0.038), and penicillin (*p* = 0.045) (Table 3).

Serotype 2 or ½ was the most frequently identified AMR pattern with resistance to 9–25 antimicrobial agents. The MDR *S. suis* strains were identified in all major serotypes, including serotype 2 or ½ (57.1%), 3 (25.0%), 8 (31.6%), 9 (68.8%), 16 (100%), 21 (87.5%), and 29 (84.2%). There were significant associations between bacterial serotypes and the susceptibility patterns toward ampicillin, cefepime, cefotaxime, ceftiofur, ceftriaxone, cefuroxime, penicillin, azithromycin, chloramphenicol, florfenicol, neomycin, spectinomycin, tylosin tartrate, enrofloxacin, levofloxacin, and trimethoprim/sulfamethoxazole (Table 4).

### 2.4. Correlations between Two Different Antibiotic Susceptibility Statuses among Isolates

**Figure 4:**
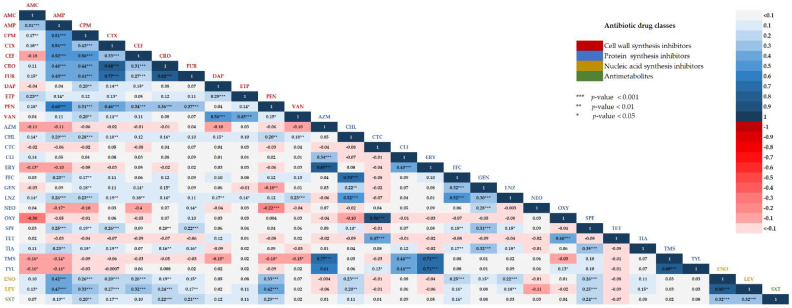
Pairwise correlation between two antimicrobial susceptibility statuses. Positive correlations are visualized in blue blocks and negative correlations in red blocks. The color intensity of the text labels is proportional to the correlation coefficients. Significant *p*-values corresponding to the correlation coefficient are indicated with asterisk (*** *p*-value < 0.001; ** *p*-value < 0.01; * *p*-value < 0.05). Cell wall synthesis inhibitor antibiotics: AMC, Amoxicillin/Clavulanic acid; AMP, Ampicillin; CEF, Ceftiofur; CPM, Cefepime; CRO, Ceftriaxone; CTX, Cefotaxime; DAP, Daptomycin; ETP, Ertapenem; FUR, Cefuroxime; MEM, Meropenem; PEN, Penicillin; VAN, Vancomycin. Protein synthesis inhibitor antibiotics: AZM, Azithromycin; NEO, Neomycin; CHL, Chloramphenicol; CLI, Clindamycin; CTC, Chlortetracycline; ERY, Erythromycin; FFC, Florfenicol; GEN, Gentamicin; LNZ, Linezolid; NEO, Neomycin; SPE, Spectinomycin; TET, Tetracycline; TMS, Tilmicosin; TYL, Tylosin tartrate; TIA, Tiamulin; OXY, Oxytetracycline. Nucleic acid synthesis inhibitor antibiotics: ENO, Enrofloxacin; LEV, Levofloxacin. Antimetabolite antibiotic: SXT, Trimethoprim/sulfamethoxazole.

The results of pairwise correlation analysis revealed varying degrees of correlation between resistance to the different antibiotics tested (Figure 4). Positive correlation refers to similarity in susceptibility or resistance of two antibiotics, while negative correlation refers to the correlation between the susceptibility of an individual drug and the resistance of another drug. Among antibiotics inhibiting cell wall synthesis, cefotaxime resistance significantly exhibited the strongest positive correlation with the resistance to ceftriaxone and cefuroxime with Pearsons’ correlation of 0.84 and 0.77, respectively (*p* < 0.001). In addition, ampicillin resistance was positively correlated with the resistance to penicillin, cefotaxime, ceftiofur, cefepime, ceftriaxone, and cefuroxime with Pearson’s correlation of 0.60, 0.54, 0.52, 0.51, 0.48, and 0.40, respectively (*p* < 0.001). In the DNA synthesis inhibitor class, enrofloxacin had the highest correlation coefficient to levofloxacin (0.80, *p* < 0.001). All cell wall synthesis and antimetabolite drugs showed a positive correlation with those of the DNA synthesis inhibitor antibiotics. A significant negative correlation was found among different drug classes, especially between protein synthesis inhibitors and cell wall synthesis inhibitors. Protein synthesis inhibitors, neomycin, were significantly negatively correlated with cell wall synthesis inhibitors, penicillin (−0.22, *p* < 0.001) and ampicillin (−0.17, *p* < 0.05). Gentamycin was also significantly negatively correlated with penicillin (−0.18, *p* < 0.01).

## 3. Discussion

*S. suis* is a major swine pathogen with the greatest impact on pig production worldwide [2]. It has considerable zoonotic potential for humans, especially in southeast Asian countries, including China, Vietnam, and Thailand [8,9,11]. Encapsulated extracellular *S. suis* is a highly invasive pathogen that causes septicemia, meningitis, endocarditis, pneumonia, and arthritis. After penetration of host mucosal barriers, it can reach and survive in the blood and finally invade multiple organs, including the lung, spleen, liver, kidney, heart, and brain [2]. In this study, a high prevalence of diseased pig-isolated *S. suis* strains was found in lung (81.7%) and brain tissues (8.1%). This data supports that the lungs and brain were major target organs for *S. suis* infection and that *S. suis* infection severely caused systemic dissemination in pigs [6].

It is known that *S. suis* serotype 2 is the most pathogenic and significantly associated with disease in both pigs and humans worldwide. However, the serotype distribution can differ over time and geographical area. In North America, multiple serotypes, such as serotypes ½, 2, 3, 8, 4, and 7, have been recorded in diseased pigs. In contrast, serotypes 2, 3, and 9 predominate in Europe and Asia [2,12]. Among diseased pigs in China, serotype 2 (66.0%) was commonly found [13], while serotype 29 (9.4%) was the most prevalent in healthy pigs, followed by serotype 2 (5.8%) and serotype 21 (4%) [9]. *S. suis* serotypes 3 (15.8%) and 2 (15.0%) were the most predominant in slaughtered and diseased pigs in South Korea [4], whereas serotype 2 (8.0%) was the most prevalent in slaughterhouse pigs in southern Vietnam [11]. Recent evidence demonstrated a higher frequency of serotype 29 among *S. suis* isolated from healthy pigs (15.4%) and pigs with respiratory disease (1.7%) in Germany [12]. In northern Thailand, serotypes 2 (19.1%) was the most common, followed by serotype 7 (15.7%), 9 (14.2%), 16 (9.3%), and 14 (7.3%) from pig tonsils at a slaughterhouse [14], while serotype 16 (11.0%) was the most frequent serotype, followed by serotypes 8 (7.0%), 9 (6.0%), and 3 (5.0%) from healthy pigs in central Thailand [15]. In agreement with previous reports, the data obtained from this study demonstrated that most *S. suis* isolates from diseased pigs were serotype 2 or ½ (25.6%), followed by serotypes 8 (9.0%) and 29 (7.1%). Serotype 29 has also been reported from *S. suis* isolated from healthy pigs in northern Thailand with a small abundance (1.0%) [16]. To the best of our knowledge, this is the first report on the high prevalence of serotype 29 identified in a large collection of *S. suis* strains isolated from diseased pigs in Thailand. The data confirmed that the distribution of different serotypes of *S. suis* in pigs could be varied by geographical localizations.

Serotypes 13, 17, and 19 have been reported from both healthy and diseased pigs, albeit with a relatively low prevalence (0.7–1.9%) [4,6,16]. However, no *S. suis* serotypes 13, 17, and 19 were found in this study, suggesting lower virulence capacities of these serotypes compared to the other common serotypes. The isolation of some uncommon *S. suis* serotypes from diseased pigs could be explained by the *S. suis* acting as an opportunistic pathogen while the inclusion of other bacterial infections being a primary cause of disease [2]. Regarding the sample sources, serotypes 2, ½, and 8 *S. suis* isolates from diseased pigs were mainly recovered from the lungs (23.4 and 9.5%, respectively), and serotype 29 was frequently isolated from the upper respiratory track (45.5%). In addition, serotype 29 was also recovered from the lung tissues (7.0%) of diseased pigs. It was possible that this serotype might be a potentially virulent serotype responsible for infections. However, virulence can also vary within serotypes [17]. Thus, further studies are needed to assess the virulence and pathogenicity of *S. suis* serotype 29. The study also revealed the dissemination of serotype 9 *S. suis* through different organs of diseased pigs, including lung, brain, and spleen, suggesting that serotype 9 is associated with invasive disease in pigs [18]. Taken together, the data suggested the dissemination of both serotype 2 and non-serotype 2 in the pig-isolated *S. suis* population in Thailand.

In southeast Asian countries, antimicrobials are freely available over the counter for use in both humans and animals, which likely contributes to the extensive use of antimicrobials in livestock sectors, leading to widespread AMR [19]. Thailand is one of the top ten veterinary antimicrobial users (4.2%) [20]. The most common drugs used are amoxicillin (39.6%), enrofloxacin (22.9%), tetracycline (12.5%), and penicillin (12.5%) [5]. For the treatment of *S. suis* infection, beta-lactams (amoxicillin/clavulanic acid, ampicillin, ceftiofur, ceftriaxone, and penicillin) and fluoroquinolones (enrofloxacin) are still the drugs of choice. However, the global trend of increasing antimicrobial resistance among streptococcal species is becoming more problematic [2].

In this study, the resistance of *S. suis* to commonly used antibiotics was relatively high. All isolates were resistant to at least one class of antibiotics, and 70.3% were resistant to three or more drug classes, which indicated substantial multidrug resistance (MDR). High frequencies of resistance were observed for protein synthesis inhibitors, such as clindamycin, tetracycline, erythromycin, and chlortetracycline, which was consistent with previous reports [6,8,9]. Among the primary drugs against *S. suis* infection, the prevalence of isolates resistant to penicillin (0–27%), ampicillin (0.6–23%), and ceftiofur (0–23%) was generally low [2,7,9]. The findings from this study are consistent with previous literature suggesting *S. suis* susceptibility to cell wall synthesis inhibitors, including beta-lactam antibiotics [6,7]. By contrast, the statistical analysis obtained from this study indicated a significant increase of antimicrobial resistance against penicillin from 47.4% to 64.3%, which was slightly higher than those from previous data in healthy pig-isolated (10.9%) and diseased pig-isolated (27.0%) *S. suis* strains in Thailand, during 2006–2007 and 2012–2015 [9]. In addition, the proportion of isolates with high penicillin MIC values increased over time, which was reflected in an increase in MIC_50_ value of 0.5 µg/mL to 2.0 µg/mL, and MIC_90_ value of >8 µg/mL. This evidence clearly confirmed the emergence and widespread nature of penicillin-resistant *S. suis* strains in Thailand. Whereas 12.6% of *S. suis* strains were resistant against the 3rd generation cephalosporin (ceftiofur), 29.7–38.1% of them were resistant to the 4th generation cephalosporin (cefepime), raising concerns that inappropriate use of cephalosporins could further accelerate widespread resistance to cephalosporins. Moreover, the presence of ampicillin resistance and the increasing prevalence of intermediate susceptible against amoxicillin/clavulanic raised awareness of the spread of resistance strains. The use of different antibiotic drugs that belong to the same categories can favor the cross-resistance of bacteria under the same resistance mechanism [21]. Recently, transferable resistance genes *cfr* and *optrA* have been identified from *S. suis* of animal origin under the selection of phenicols and other ribosomal-targeted antibiotics, which are broadly used in veterinary medicine [8,21]. However, these resistance genes were not only associated with resistance to phenicols but also conferred resistance to oxazolidinone (linezolid and tedizolid), available antibiotic drugs used only in humans. This evidence suggests the impact of antibiotic-resistant selection on farms to human health. In this study, the emergence of pig-isolated *S. suis* strains resistant to drugs used for humans, such as linezolid, vancomycin, and meropenem, was found. This finding raised serious concern about the transmission of antibiotic-resistant *S. suis* strains among animals and humans, causing clinical or epidemiological problems in the near future. The overuse of different antimicrobial substances in pig farming could induce more variation of antimicrobial resistance in *S. suis* of human origin. Therefore, proper use of antibiotic drugs for prophylaxis and treatment in swine production systems is highly recommended to avoid further spread of AMR *S. suis* in both animals and humans.

A remarkably high prevalence of *S. suis* isolates resistant to antibiotics inhibiting protein synthesis was determined in this study. A high prevalence of tetracycline-, macrolide-, and lincosamide-resistant *S. suis* isolates was observed in various countries in Asia, Europe, North America, and Africa [2,7,9]. Such great resistance is undoubtedly related to their intensive use in swine industries, and it could be associated with the acquisition and dissemination of AMR genes through mobile-genetic elements (MGEs). The acquisition and dissemination of AMR genes in streptococci is strongly associated with MGEs, mainly integrative and conjugative elements (ICEs) and prophages. A variety of AMR determinants for tetracyclines [*tet*(M), *tet*(L), *tet*(O), and *tet*(40)], macrolides [*erm*(B)], aminoglycosides (*aph*A3, *sat*, *ant*6, and *aad*E), and phenicols (*cat*) have been located in the ICE*Sa*2603 family [22]. For fluoroquinolones, the prevalence of enrofloxacin- and levofloxacin-resistant *S. suis* isolates was significant. A high frequency of intermediate susceptibility to levofloxacin (45.2–54.3%), suggesting the continued use of fluoroquinolones, could eventually lead to the emergence of resistance. In addition, resistance to macrolide and fluoroquinolone drugs could immensely limit the therapeutic use of these antibiotics for the treatment of *S. suis* infection. A more comprehensive investigation and characterization of the genetic determinants and understanding of the AMR mechanisms of *S. suis* strains in Thailand are needed for effective monitoring and preventing the spread of AMR in this region.

The AMR problem drastically impairs the effectiveness of the therapeutic use of existing antibiotics. Antibiotic combination therapy with different modes of action is a far more effective approach for combating MDR pathogens and preventing the emergence of resistance commonly found with monotherapy [23]. The combination of beta-lactams with gentamicin, displaying a strong synergistic effect against *Streptococcus pneumoniae* infection, has been reported [24]. In addition, Yu et al. [25] demonstrated a marked synergistic activity of the two combination regimens, including ampicillin plus apramycin and tiamulin plus spectinomycin, for treatment of *S. suis* infection. In this study, a significant negative correlation was found among different drug classes, especially between cell wall synthesis inhibitors, penicillin, and protein synthesis inhibitors, including neomycin, and gentamicin. Combination therapy of penicillin or ampicillin plus neomycin or gentamicin may be used as a treatment option for *S. suis* infection. For further study, to determine the effectiveness of combined drugs used against zoonotic *S. suis* infection, investigation, and verification of possible drug combinations should be performed for both animal and human antimicrobial agents.

## 4. Materials and Methods

### 4.1. Bacterial Collection

A total of 246 non-duplicate *S. suis* strains were collected from specimens (organs, tissues, and swabs) of diseased pigs across Thailand from 2018–2020 as a part of routine laboratory tests at the Veterinary Diagnostic Laboratory, Large Animal Teaching Hospital, Faculty of Veterinary Science, Chulalongkorn University. The *S. suis* strains were isolated on Columbia blood agar (5% sheep blood) at 37 °C in 5% CO_2_ for 24–48 h. The isolates with alpha hemolytic colonies were further identified by conventional biochemical tests [26]. Subsequently, the colonies were confirmed to be *S. suis* by the PCR-based approach targeting the glutamate dehydrogenase (*gdh*) gene [3] and the recombination/repair protein (*recN*) gene. The PCR primers used for *recN* identification were SuisRecNsy01_F (5′-TTA TCT GTC TTG AAA CAG ATT GGG-3′) and SuisRecNsy01_R (5′-TCT TTC TCT AAG TTC TTA AGC TGA AC-3′). The PCR conditions comprised an initial denaturation step at 95 °C for 5 min, followed by 35 cycles of 95 °C for 30 s, 54 °C for 1 min, and 72 °C for 1 min, with a final extension step at 72 °C for 7 min.

### 4.2. Multiplex PCR-Based Serotyping

Identification of *S. suis* serotypes was conducted using a multiplex PCR-based method [3]. The PCR reactions were carried out independently in four sets; the first set included primers for serotypes ½, 1, 2, 3, 7, 9, 11, 14, and 16; the second for serotypes 4, 5, 8, 12, 18, 19, 24, and 25; the third for serotypes 6, 10, 13, 15, 17, 23, and 31; and the fourth for serotypes 21, 27, 28, 29, and 30. PCR amplification of the *S. suis* species-specific PCR targeting the *gdh* gene was also carried out as a positive control of the reaction. The oligonucleotide primer sequences are listed in Table S7. The following PCR conditions were used: initial denaturation at 95 °C for 3 min, followed by 30 cycles of 95 °C for 20 s, 62 °C for 1.30 min, and 62 °C for 1.30 min, and a final extension at 72 °C for 5 min.

### 4.3. Antimicrobial Susceptibility Testing

The MICs of different antimicrobial agents were determined by the broth microdilution method using a semi-automatic system (Sensititre, Trek Diagnostic Systems Ltd., West Sussex, UK) in accordance with the Clinical and Laboratory Standards Institute (CLSI) recommendations [27]. The MIC test was performed with two sets of commercially prepared, dehydrated 96-well microtiter plates, including Sensititre Vet Bovine/Swine BOPO6F plate and Sensititre Streptococcus species STP6F plate, containing antibiotics for veterinary and human usages, respectively. A total of 35 antibiotics from different drug classes and mechanisms of action were included in this study (Table S3). The MIC test conditions were performed according to the manufacturer’s guidelines with minor modifications. In brief, isolates were cultured on Columbia blood agar at 37 °C in 5% CO_2_ incubator overnight. Selected colonies were suspended in Sensititre cation-adjusted Mueller-Hinton broth (CAMHBT) and adjusted to be a 0.5 McFarland standard. Subsequently, a 100-µL aliquot of the suspension was transferred into a tube of CAMHBT and CAMHBT with lysed Horse blood (CAMHBT+LHB) for BOPO6F and STP6F panels, respectively, to obtain an inoculum density of 5 × 10^5^ CFU/mL. The BOPO6F and STP6F panels were reconstituted by adding 50 µL and 100 µL/well, respectively, and the plates were covered with an adhesive seal and incubated at 35 ± 2 °C in Sensititre ARISTM 2X for 20–24 h. The MIC value, the lowest drug concentration inhibiting visible growth, was read automatically on the Sensititre ARISTM 2X and read visually using a manual viewbox according to the instructions in Sensititre SWIN software.

*Streptococcus pneumoniae* ATCC 49619, *Staphylococcus aureus* ATCC 29213, *Escherichia coli* ATCC 25922, and *S. suis* serotype 2-P1/7 (UK) were used as control and reference strains, and MICs were within the accepted quality control ranges. The results were interpreted according to CLSI veterinary breakpoints [28], EUCAST [29], FDA [30], and previously reported data when available (Table S3).

### 4.4. Statistical Analysis

Statistical differences were determined by performing chi-square tests with the STATA statistical package v14.0 (Stata Corporation, College Station, TX, USA). Pairwise analysis of the correlation between the antimicrobial susceptibility status (susceptible, intermediate, and resistant) to the different antibiotics was investigated using Pearson’s correlation analysis. A *p*-value of <0.05 was considered to be statistically significant.

## 5. Conclusions

The predominance of serotype 2 or ½, followed by 8, 29, 9, and 21, was identified from diseased pig-isolated *S. suis* strains in different regions of Thailand from 2018–2020. To the best of our knowledge, this is the first report describing the distribution of *S. suis* serotype 29 in a large population of diseased pigs. The surveillance of antimicrobial resistance confirmed widespread AMR and MDR *S. suis* strains against different commonly available antibiotic drug classes. Although drugs inhibiting cell wall synthesis were the most effective antibiotics, a tendency toward reduced efficacy of these drugs was observed. In addition, the proportion of intermediate susceptibility and resistance to many antibiotics increased over time. As a result, effective antibiotic drugs for the treatment of *S. suis* infection in both animals and humans could be limited in the near future. In this study, pairwise correlation between two antimicrobial susceptibility statuses suggested that the combination of cell wall synthesis inhibitors (penicillin) with protein synthesis inhibitors (neomycin and gentamicin) may be used as a choice for treatment of *S. suis* infection; this therapeutic approach deserves additional study. Taken together, the knowledge gained from this study underlined the resistance selective pressure in livestock systems, raising an awareness of prudent and efficient use of therapeutic options for *S. suis* infection in both public and veterinary healthcare. Continuous surveillance is required to monitor the prevalence of AMR and MDR in *S. suis* and to guide decisions regarding appropriate treatment. Further research focusing on the understanding of AMR mechanisms would be helpful and necessary for developing effective preventive measures for *S. suis* infection. In addition, effective prevention and infection control strategies should be made to prevent the dissemination of AMR and MDR in *S. suis* in the country.

## Figures and Tables

**Table 1:** Distribution of *S. suis* strains according to serotype and isolation period.

Serotypes	Year of Isolation, *n* (%)			*p*-Values	Total
	2018 *n* = 72	2019 *n* = 97	2020 *n* = 77		
Serotype 2 or ½	18 (25.0)	26 (26.8)	19 (24.7)	0.941	63 (25.6)
Serotype 3	4 (5.6)	4 (4.1)	4 (5.2)	0.902	12 (4.9)
Serotype 8	4 (5.6)	11 (11.3)	4 (5.2)	0.229	19 (7.7)
Serotype 9	3 (4.2)	6 (6.2)	7 (9.1)	0.470	16 (6.5)
Serotype 16	4 (5.6)	2 (2.1)	3 (3.9)	0.485	9 (3.7)
Serotype 21	3 (4.2)	7 (7.2)	6 (7.8)	0.626	16 (6.5)
Serotype 29	4 (5.6)	7 (7.2)	8 (10.4)	0.528	19 (7.7)
Other serotypes ^a^	21 (29.2)	21 (21.6)	16 (20.8)	0.410	58 (23.6)
Non-typeable	11 (15.3)	13 (13.4)	10 (13.0)	0.910	34 (13.8)

^a^ Other serotypes, including serotype 1 or 14 (*n* = 6), 4 (*n* = 6), 5 (*n* = 8), 6 (*n* = 1), 7 (*n* = 5), 10 (*n* = 1), 11 (*n* = 1), 12 (*n* = 1), 15 (*n* = 2), 18 (*n* = 6), 23 (*n* = 1), 24 (*n* = 2), 25 (*n* = 1), 27 (*n* = 4), 28 (*n* = 4), 30 (*n* = 1), and 31 (*n* = 8).

**Table 2:** Minimum inhibitory concentration (MIC) values distribution, MIC_50_ and MIC_90_ values, and resistance rates of *S. suis* strains from 2018–2020.

Antibiotic Drugs	MIC Breakpoints (µg/mL)			MIC Values (µg/mL) ^a^																	MIC_50_	MIC_90_	S (%)	I (%)	R (%)	MIC Ranges
	S	I	R	0.008	0.016	0.03	0.06	0.125	0.25	0.5	1	2	4	8	16	32	64	128	256	512						
Amoxicillin/Clavulanic acid	≤8/4	16/8	≥32/16									194	13	27	8	4					≤2	8	95.1	3.3	1.6	≤2–>16
Ampicillin	≤0.5	1	≥2						150	11	6	15	13	7	13	31					≤0.25	>16	65.4	2.4	32.1	≤0.25–>16
Cefepime	≤2	4	≥8							168	26	18	18	10	6						≤0.5	4	68.3	ND	31.7	≤0.5–>8
Cefotaxime	≤0.5	-	≥1					27	20	52	64	28	12	43							1	>4	40.2	ND	59.8	≤0.12–>4
Ceftiofur	≤0.5	-	≥1						142	24	29	15	5	15	16						≤0.25	8	85.4	2.0	12.6	≤0.25–>8
Ceftriaxone	≤0.5	-	≥1					30	16	46	68	22	64								1	>2	37.4	ND	62.6	≤0.12–>2
Cefuroxime	≤0.5	-	≥1							79	76	38	7	46							1	>4	32.1	ND	67.9	≤0.5–>4
Daptomycin	≤1	-	≥2				22	98	107	6	6	1	6								0.25	0.25	97.2	ND	2.8	≤0.06–>2
Ertapenem	≤0.5	-	≥1							240	3	2	1								≤0.5	≤0.5	97.6	ND	2.4	≤0.5–4
Meropenem	≤2	-	≥4						241	4	1										≤0.25	≤0.25	100.0	ND	0.0	≤0.25–1
Penicillin	≤0.25	0.5	≥1			15	10	20	28	41	19	16	27	16	54						1	>8	29.7	16.7	53.7	≤0.03–>8
Vancomycin	≤1	-	≥2							237	2	3	1	3							≤0.5	≤0.5	97.2	ND	2.8	≤0.5–>4
Azithromycin	≤0.5	1	≥2						7	1	1	2	235								>2	>2	3.3	0.4	96.3	≤0.25–>2
Chloramphenicol	≤4	8	≥16									5	64	72	41	38	26				8	>32	28.0	29.3	42.7	2–>32
Chlortetracycline	≤2	4	≥8							4	4		3	17	218						>8	>8	3.3	1.2	95.5	≤0.5–>8
Clindamycin	≤0.5	1–2	≥4						1				2	2	3	238					>16	>16	0.4	0.0	99.6	0.25–>16
Erythromycin	≤0.25	0.5	≥1						6	1	3	6	230								>2	>2	2.4	0.4	97.2	≤0.25–>2
Florfenicol	≤2	4	≥8								6	58	58	14	110						4	>8	26.0	23.6	50.4	1–>8
Gentamicin	≤4	8	≥16								24	35	47	16	11	113					8	>16	43.1	6.5	50.4	≤1–>16
Linezolid	≤2	-	≥4						2	25	98	56	52	13							1	4	73.6	ND	26.4	≤0.25–>4
Neomycin	≤16	-	≥32										26	70	51	33	66				16	>32	59.8	ND	40.2	≤4–>32
Oxytetracycline	≤4	-	≥8							3	2	4		6	231						>8	>8	3.7	ND	96.3	≤0.5–>8
Spectinomycin	≤64	-	≥128											17	46	40	7	136			>64	>64	44.7	ND	55.3	≤8–>64
Tetracycline	≤0.5	1	≥2								2	3		2	239						>8	>8	0.0	0.8	99.2	≤1–>8
Tiamulin	≤16	-	≥32							9	8	12	2	7	13	7	188				>32	>32	20.7	ND	79.3	≤0.5–>32
Tigecycline	≤0.25	-	≥0.5		2	19	53	68	104												0.12	>0.12	ND	ND	ND	≤0.02–>0.12
Tilmicosin	≤16	-	≥32										3	2		1		240			>64	>64	2.0	ND	98.0	≤4–>64
Tulathromycin	ND	ND	ND								1	3	2		2	2	4	232			>64	>64	ND	ND	ND	≤1–>64
Tylosin tartrate	≤4	-	≥8								4	1				1	240				>32	>32	2.0	ND	98.0	1–>32
Danofloxaci	ND	ND	ND					2	8	51	53	132									>1	>1	ND	ND	ND	≤0.12–>1
Enrofloxacin	≤0.5	1	≥2					1	20	70	20	12	123								2	>2	37.0	8.1	54.9	≤0.12–>2
Levofloxacin	≤0.01	0.03–2	≥4							83	32	8	12	111							2	>4	0.0	50.0	50.0	≤0.5–>4
Moxifloxacin	≤0.5	-	≥1								206	27	12	1							≤1	2	ND	ND	ND	≤1–8
Sulphadimethoxine	ND	ND	ND																13	233	>256	>256	ND	ND	ND	≤256–>256
Trimethoprim/sulfamethoxazole	≤0.5/9.5	1/19–2/38	≥4/76							79	5	3	26	133							>2	>4	32.1	3.3	64.6	≤0.5–>2

^a^ White cells indicate the dilution range tested. Green and red vertical lines, respectively, describe the susceptible and resistant clinical breakpoints recommended by the CLSI (Vet01S, 2020), EUCAST (EUCAST, 2020), FDA (FDA, 2019), and previously reported data. MIC, minimum inhibitory concentration values, which are interpreted as susceptible (S), intermediate (I), and resistant (R). MIC_50_, the MIC that inhibits 50% of the isolates tested; MIC_90_, the MIC that inhibits 90% of the isolates tested; ND, no data/not determined.

**Table 3:** Antimicrobial susceptibility of *S. suis* strains according to their isolation period.

Antibiotic Drugs	Antimicrobial Susceptibility, *n* (%)									*p*-Values
	2018 *n* = 72			2019 *n* = 97			2020 *n* = 77			
	S	I	R	S	I	R	S	I	R	
Amoxicillin/Clavulanic acid	67 (93.1)	1 (1.4)	4 (5.6)	94 (96.9)	3 (3.1)	0 (0.0)	73 (94.8)	4 (5.2)	0 (0.0)	0.022 *
Ampicillin	56 (77.8)	1 (1.4)	15 (20.8)	61 (62.9)	1 (1.0)	35 (36.1)	44 (57.1)	4 (5.2)	29 (37.7)	0.038 *
Cefepime	53 (73.6)	ND	19 (26.4)	69 (71.1)	ND	28 (28.9)	46 (59.7)	ND	31 (40.3)	0.142
Cefotaxime	28 (38.9)	ND	44 (61.1)	41 (42.3)	ND	56 (57.7)	30 (39.0)	ND	47 (61.0)	0.872
Ceftiofur	65 (90.3)	0 (0.0)	7 (9.7)	83 (85.6)	3 (3.1)	11 (11.3)	62 (80.5)	2 (2.6)	13 (16.9)	0.373
Ceftriaxone	26 (36.1)	ND	46 (63.9)	40 (41.2)	ND	57 (58.8)	26 (33.8)	ND	51 (66.2)	0.578
Cefuroxime	23 (31.9)	ND	49 (68.1)	37 (38.1)	ND	60 (61.9)	19 (24.7)	ND	58 (75.3)	0.168
Daptomycin	69 (95.8)	ND	3 (4.2)	95 (97.9)	ND	2 (2.1)	75 (97.4)	ND	2 (2.6)	0.709
Ertapenem	70 (97.2)	ND	2 (2.8)	95 (97.9)	ND	2 (2.1)	75 (97.4)	ND	2 (2.6)	0.951
Meropenem	72 (100.0)	ND	0 (0.0)	97 (100.0)	ND	0 (0.0)	77 (100.0)	ND	0 (0.0)	1.000
Penicillin	28 (38.9)	10 (13.9)	34 (47.2)	24 (24.7)	23 (23.7)	50 (51.5)	21 (27.3)	8 (10.4)	48 (62.3)	0.045 *
Vancomycin	71 (98.6)	ND	1 (1.4)	93 (95.9)	ND	4 (4.1)	75 (97.4)	ND	2 (2.6)	0.565
Azithromycin	2 (2.8)	0 (0.0)	70 (97.2)	3 (3.1)	1 (1.0)	93 (95.9)	3 (3.9)	0 (0.0)	74 (96.1)	0.791
Chloramphenicol	16 (22.2)	28 (38.9)	28 (38.9)	34 (35.1)	21 (21.6)	42 (43.3)	19 (24.7)	23 (29.9)	35 (45.5)	0.113
Chlortetracycline	2 (2.8)	2 (2.8)	68 (94.4)	3 (3.1)	1 (1.0)	93 (95.9)	2 (2.6)	0 (0.0)	75 (97.4)	0.588
Clindamycin	1 (1.4)	0 (0.0)	71 (98.6)	0 (0.0)	0 (0.0)	97 (100.0)	0 (0.0)	0 (0.0)	77 (100.0)	0.297
Erythromycin	2 (2.8)	0 (0.0)	70 (97.2)	2 (2.1)	1 (1.0)	94 (96.9)	2 (2.6)	0 (0.0)	75 (97.4)	0.802
Florfenicol	20 (27.8)	20 (27.8)	32 (44.4)	29 (29.9)	18 (18.6)	50 (51.5)	15 (19.5)	20 (26.0)	42 (54.5)	0.346
Gentamicin	35 (48.6)	3 (4.2)	34 (47.2)	38 (39.2)	7 (7.2)	52 (53.6)	33 (42.9)	6 (7.8)	38 (49.4)	0.719
Linezolid	55 (76.4)	ND	17 (23.6)	72 (74.2)	ND	25 (25.8)	54 (70.1)	ND	23 (29.9)	0.676
Neomycin	38 (52.8)	ND	34 (47.2)	63 (64.9)	ND	34 (35.1)	46 (59.7)	ND	31 (40.3)	0.280
Oxytetracycline	2 (2.8)	ND	70 (97.2)	5 (5.2)	ND	92 (94.8)	2 (2.6)	ND	75 (97.4)	0.600
Spectinomycin	37 (51.4)	ND	35 (48.6)	45 (46.4)	ND	52 (53.6)	28 (36.4)	ND	49 (63.6)	0.167
Tetracycline	0 (0.0)	0 (0.0)	72 (100.0)	0 (0.0)	2 (2.1)	95 (97.9)	0 (0.0)	0 (0.0)	77 (100.0)	0.213
Tiamulin	19 (26.4)	ND	53 (73.6)	18 (18.6)	ND	79 (81.4)	14 (18.2)	ND	63 (81.8)	0.370
Tilmicosin	2 (2.8)	ND	70 (97.2)	1 (1.0)	ND	96 (99.0)	2 (2.6)	ND	75 (97.4)	0.666
Tylosin tartrate	2 (2.8)	ND	70 (97.2)	2 (2.1)	ND	95 (97.9)	1 (1.3)	ND	76 (98.7)	0.815
Enrofloxacin	23 (31.9)	7 (9.7)	42 (58.3)	38 (39.2)	9 (9.3)	50 (51.5)	30 (39.0)	4 (5.2)	43 (55.8)	0.687
Levofloxacin	0 (0.0)	39 (54.2)	33 (45.8)	0 (0.0)	47 (48.5)	50 (51.5)	0 (0.0)	37 (48.1)	40 (51.9)	0.701
Trimethoprim/sulfamethoxazole	26 (36.1)	3 (4.2)	43 (59.7)	35 (36.1)	3 (3.1)	59 (60.8)	19 (24.7)	2 (2.6)	56 (72.7)	0.495

ND: no data/not determined; S: susceptible; I: intermediate; R: resistant; * *p*-value < 0.05.

**Table 4:** Antimicrobial susceptibility of *S. suis* strains according to serotype.

Antibiotic Drugs	Antimicrobial Susceptible, *n* (%)									*p*-Values
	Serotype 2 or ½	Serotype 3	Serotype 8	Serotype 9	Serotype 16	Serotype 21	Serotype 29	Other Serotypes ^a^	Non- Typeable	
	*n* = 63	*n* = 12	*n* = 19	*n* = 16	*n* = 9	*n* = 16	*n* = 19	*n* = 58	*n* = 34	
Amoxicillin/Clavulanic Acid	62 (98.4)	12 (100.0)	19 (100.0)	16 (100.0)	9 (100.0)	15 (93.8)	17 (89.5)	55 (94.8)	29 (85.3)	0.566
Ampicillin	50 (79.4)	12 (100.0)	17 (89.5)	12 (75.0)	4 (44.4)	10 (62.5)	8 (42.1)	38 (65.5)	10 (29.4)	<0.001 *
Cefepime	52 (82.5)	11 (91.7)	17 (89.5)	12 (75.0)	3 (33.3)	11 (68.8)	13 (68.4)	36 (62.1)	13 (38.2)	<0.001 *
Cefotaxime	24 (38.1)	6 (50.0)	16 (84.2)	8 (50.0)	4 (44.4)	6 (37.5)	6 (31.6)	24 (41.4)	5 (14.7)	0.001 *
Ceftiofur	59 (93.7)	12 (100.0)	18 (94.7)	16 (100.0)	6 (66.7)	12 (75.0)	16 (84.2)	49 (84.5)	22 (64.7)	0.013 *
Ceftriaxone	23 (36.5)	6 (50.0)	16 (84.2)	6 (37.5)	3 (33.3)	6 (37.5)	6 (31.6)	19 (32.8)	7 (20.6)	0.003 *
Cefuroxime	19 (30.2)	5 (41.7)	15 (78.9)	3 (18.8)	4 (44.4)	5 (31.3)	3 (15.8)	19 (32.8)	6 (17.6)	0.001 *
Daptomycin	61 (96.8)	12 (100.0)	18 (94.7)	16 (100.0)	9 (100.0)	14 (87.5)	19 (100.0)	57 (98.3)	33 (97.1)	0.461
Ertapenem	62 (98.4)	12 (100.0)	19 (100.0)	16 (100.0)	9 (100.0)	15 (93.8)	18 (94.7)	58 (100.0)	31 (91.2)	0.233
Meropenem	63 (100.0)	12 (100.0)	19 (100.0)	16 (100.0)	9 (100.0)	16 (100.0)	19 (100.0)	58 (100.0)	34 (100.0)	ND
Penicillin	30 (47.6)	9 (75.0)	8 (42.1)	5 (31.3)	3 (33.3)	3 (18.8)	0 (0.0)	13 (22.4)	2 (5.9)	<0.001 *
Vancomycin	61 (96.8)	12 (100.0)	19 (100.0)	16 (100.0)	9 (100.0)	14 (87.5)	19 (100.0)	57 (98.3)	32 (94.1)	0.341
Azithromycin	1 (0.02)	0 (0.0)	1 (5.3)	0 (0.0)	2 (22.2)	2 (12.5)	2 (10.5)	0 (0.0)	0 (0.0)	0.025*
Chloramphenicol	30 (47.6)	1 (8.3)	2 (10.5)	7 (43.8)	1 (11.1)	7 (43.8)	8 (42.1)	9 (15.5)	4 (11.8)	<0.001 *
Chlortetracycline	1 (1.6)	1 (8.3)	0 (0.0)	1 (6.3)	0 (0.0)	0 (0.0)	1 (5.3)	4 (6.9)	0 (0.0)	0.256
Clindamycin	0 (0.0)	0 (0.0)	1 (5.3)	0 (0.0)	0 (0.0)	0 (0.0)	0 (0.0)	0 (0.0)	0 (0.0)	0.151
Erythromycin	1 (1.6)	0 (0.0)	1 (5.3)	0 (0.0)	1 (11.1)	1 (6.3)	2 (10.5)	0 (0.0)	0 (0.0)	0.264
Florfenicol	19 (30.2)	2 (16.7)	3 (15.8)	7 (43.8)	1 (11.1)	6 (37.5)	8 (42.1)	11 (19.0)	7 (20.6)	0.012 *
Gentamicin	19 (30.2)	5 (41.7)	9 (47.4)	7 (43.8)	2 (22.2)	6 (37.5)	12 (63.2)	33 (56.9)	13 (38.2)	0.078
Linezolid	47 (74.6)	8 (66.7)	12 (63.2)	15 (93.8)	6 (66.7)	9 (56.3)	15 (78.9)	47 (81.0)	22 (64.7)	0.216
Neomycin	27 (42.9)	7 (58.3)	11 (57.9)	8 (50.0)	4 (44.4)	7 (43.8)	16 (84.2)	41 (70.7)	26 (76.5)	0.004 *
Oxytetracycline	1 (1.6)	1 (8.3)	1 (5.3)	1 (6.3)	0 (0.0)	0 (0.0)	1 (5.3)	4 (6.9)	0 (0.0)	0.641
Spectinomycin	23 (36.5)	7 (58.3)	14 (73.7)	9 (56.3)	3 (33.3)	5 (31.3)	10 (52.6)	28 (48.3)	11 (32.4)	0.071
Tetracycline	0 (0.0)	0 (0.0)	0 (0.0)	0 (0.0)	0 (0.0)	0 (0.0)	0 (0.0)	0 (0.0)	0 (0.0)	0.978
Tiamulin	11 (17.5)	5 (41.7)	4 (21.1)	2 (12.5)	0 (0.0)	4 (25.0)	6 (31.9)	15 (25.9)	4 (11.8)	0.216
Tilmicosin	1 (1.6)	0 (0.0)	1 (5.3)	0 (0.0)	0 (0.0)	1 (6.3)	2 (10.5)	0 (0.0)	0 (0.0)	0.149
Tylosin tartrate	1 (1.6)	0 (0.0)	1 (5.3)	1 (6.3)	0 (0.0)	0 (0.0)	2 (10.5)	0 (0.0)	0 (0.0)	0.149
Enrofloxacin	27 (42.9)	9 (75.0)	9 (47.4)	11 (68.8)	1 (11.1)	2 (12.5)	6 (31.6)	21 (36.2)	5 (14.7)	<0.001 *
Levofloxacin	0 (0.0)	0 (0.0)	0 (0.0)	0 (0.0)	0 (0.0)	0 (0.0)	0 (0.0)	0 (0.0)	0 (0.0)	<0.001 *
Trimethoprim/sulfamethoxazole	28 (44.4)	10 (83.3)	12 (63.2)	6 (37.5)	0 (0.0)	3 (18.8)	1 (5.3)	13 (22.4)	6 (17.6)	<0.001 *

^a^ Other serotypes including serotype 1 or 14 (*n* =6), 4 (*n* = 6), 5 (*n* = 8), 6 (*n* =1), 7 (*n* = 5), 10 (*n* =1), 11 (*n* = 1), 12 (*n* = 1), 15 (*n* = 2), 18 (*n* = 6), 23 (*n* = 1), 24 (*n* = 2), 25 (*n* = 1), 27 (*n* = 4), 28 (*n* = 4), 30 (*n* = 1), and 31 (*n* = 8). * *p*-value < 0.05.

## Data Availability

Not applicable.

## Supplementary Materials

The following supporting information can be downloaded at: https://www.mdpi.com/article/10.3390/antibiotics11030410/s1, Table S1: Sources of *S. suis* strains isolated from diseased pigs from 2018–2020; Table S2: Distribution of *S. suis* serotypes in different sources of specimens; Table S3: MIC breakpoints and interpretative categories of antimicrobial susceptibility test; Table S4: Minimum inhibitory concentration (MIC) value distribution, MIC_50_ and MIC_90_ values, and resistance rates of 72 *S. suis* strains in 2018; Table S5: Minimum inhibitory concentration (MIC) value distribution, MIC_50_ and MIC_90_ values, and resistance rates of 97 *S. suis* strains in 2019; Table S6: Minimum inhibitory concentration (MIC) value distribution, MIC_50_ and MIC_90_ values, and resistance rates of 77 *S. suis* strains in 2020; Table S7: Oligonucleotide primer sequences.
