# Supplementary material for: Antimicrobial Susceptibility of Streptococcus suis Isolated from Diseased Pigs in Thailand, 2018–2020
Source: Antibiotics (Basel). 2022 Mar 18;11(3):410. doi: 10.3390/antibiotics11030410 (PMC8944821; doi:10.3390/antibiotics11030410)
Supplement: Supplementary file 1 [file antibiotics-11-00410-s001.zip › antibiotics-1612682-supplementary.pdf]

Table S1. Sources of *S. suis* strains isolated from diseased pigs during 2018-2020.

Provinces	Farms	Strains	Sources of specimens, n (%)								
			Blood	Brain	Joint fluid	Lung	Nasal swab	Pleural effusion	Spleen	Tongue swab	Vaginal swab
n=14	n=105	n=246	n=2	n=20	n=6	n=201	n=11	n=1	n=2	n=1	n=2
Nakhon Pathom	33 (31.4)	84 (34.1)	1 (50.0)	4 (20.0)	-	76 (37.8)	2 (18.2)	-	1 (50.0)	-	-
Ratchaburi	32 (30.5)	77 (31.3)	-	7 (35.0)	4 (66.7)	59 (29.4)	4 (36.4)	1 (100.0)	-	-	2 (100.0)
Chon Buri	13 (12.4)	35 (14.2)	-	-	-	31 (15.4)	3 (27.3)	-	1 (50.0)	-	-
Chachoengsao	6 (5.7)	13 (5.3)	-	2 (10.0)	-	10 (5.0)	-	-	-	1 (100.0)	-
Lopburi	4 (3.8)	17 (6.9)	-	5 (25.0)	1 (16.7)	9 (4.5)	2 (18.2)	-	-	-	-
Prachin Buri	3 (2.9)	4 (1.6)	-	-	-	4 (2.0)	-	-	-	-	-
Kanchanaburi	3 (2.9)	3 (1.2)	-	-	-	3 (1.5)	-	-	-	-	-
Suphan Buri	3 (2.9)	3 (1.2)	-	-	-	3 (1.5)	-	-	-	-	-
Khon Kaen	2 (1.9)	4 (1.6)	-	2 (10.0)	1 (16.7)	1 (0.5)	-	-	-	-	-
Nakhon Ratchasima	2 (1.9)	2 (0.8)	-	-	-	2 (1.0)	-	-	-	-	-
Nakhon Sawan	1 (1.0)	1 (0.4)	-	-	-	1 (0.5)	-	-	-	-	-
Phuket	1 (1.0)	1 (0.4)	-	-	-	1 (0.5)	-	-	-	-	-
Saraburi	1 (1.0)	1 (0.4)	1 (50.0)	-	-	-	-	-	-	-	-
Ubon Ratchathani	1 (1.0)	1 (0.4)	-	-	-	1 (0.5)	-	-	-	-	-

Table S2. Distribution of *S. suis* serotypes in different sources of specimens.

Serotypes	Strains	Sources of specimens, n (%)								
		Blood	Brain	Joint fluid	Lung	Nasal swab	Pleural effusion	Spleen	Tongue swab	Vaginal swab
	n=246	n=2	n=20	n=6	n=201	n=11	n=1	n=2	n=1	n=2
1 or 14	6 (2.4)	-	1 (5.0)	1 (16.7)	4 (2.0)	-	-	-	-	-
2 or ½	63 (25.6)	1 (50.0)	10 (50.0)	3 (50.0)	47 (23.4)	1 (9.1)	-	1 (50.0)	-	-
3	12 (4.9)	-	-	1 (16.7)	11 (5.5)	-	-	-	-	-
4	6 (2.4)	-	2 (10.0)	-	4 (2.0)	-	-	-	-	-
5	8 (3.3)	-	-	-	8 (4.0)	-	-	-	-	-
6	1 (0.4)	-	-	-	1 (0.5)	-	-	-	-	-
7	5 (2.0)	-	-	-	5 (2.5)	-	-	-	-	-
8	19 (7.7)	-	-	-	19 (9.5)	-	-	-	-	-
9	16 (6.5)	-	6 (30.0)	-	9 (4.5)	-	1 (100.0)	-	-	-
10	1 (0.4)	-	-	-	1 (0.5)	-	-	-	-	-
11	1 (0.4)	-	-	-	1 (0.5)	-	-	-	-	-
12	1 (0.4)	-	-	-	1 (0.5)	-	-	-	-	-
15	2 (0.8)	-	-	-	2 (1.0)	-	-	-	-	-
16	9 (3.7)	-	-	-	6 (3.0)	2 (18.2)	-	-	-	1 (50.0)
18	6 (2.4)	-	1 (5.0)	1 (16.7)	4 (2.0)	-	-	-	-	-
21	16 (6.5)	-	-	-	14 (7.0)	2 (18.2)	-	-	-	-
23	1 (0.4)	-	-	-	1 (0.5)	-	-	-	-	-
24	2 (0.8)	-	-	-	2 (1.0)	-	-	-	-	-
25	1 (0.4)	-	-	-	1 (0.5)	-	-	-	-	-
27	4 (1.6)	-	-	-	4 (2.0)	-	-	-	-	-
28	4 (1.6)	-	-	-	2 (1.0)	1 (9.1)	-	-	1 (100.0)	-
29	19 (7.7)	-	-	-	14 (7.0)	5 (45.5)	-	-	-	-
30	1 (0.4)	-	-	-	1 (0.5)	-	-	-	-	-
31	8 (3.3)	-	-	-	7 (3.5)	-	-	-	-	1 (50.0)
Non-typeable	34 (13.8)	1 (50.0)	-	-	32 (15.9)	-	-	1 (50.0)	-	-

Table S3. MIC breakpoints and interpretative categories of antimicrobial susceptibility test.

Antibiotic drugs			Interpretive categories and MIC breakpoints, μg/mL [a]			References
			Susceptibility (S)	Intermediate (I)	Resistance (R)	
Cell-wall synthesis inhibitors	AMC	Amoxicillin/Clavulanicacid	≤ 8/4	16/8	≥ 32/16	Hernandez-Garcia et al., 2017
	AMP	Ampicillin	≤ 0.5	1	≥ 2	CLSI Vet01S, 2020
	CPM	Cefepime	≤ 0.5	-	≥ 1	EUCAST, 2020
	CTX	Cefotaxime	≤ 0.5	-	≥ 1	EUCAST, 2020
	CEF	Ceftiofur	≤ 2	4	≥ 8	CLSI Vet01S, 2020
	CRO	Ceftriaxone	≤ 0.5	-	≥ 1	EUCAST, 2020
	FUR	Cefuroxime	≤ 0.5	-	≥ 1	EUCAST, 2020
	DAN	Danofloxacin	ND	ND	ND	ND
	DAP	Daptomycin	≤ 1	-	≥ 2	CLSI M100, 2020
	ETP	Ertapenem	≤ 0.5	-	≥ 1	EUCAST, 2020
	MEM	Meropenem	≤ 2	-	≥ 4	EUCAST, 2020
	PEN	Penicillin	≤ 0.25	0.5	≥ 1	CLSI Vet01S, 2020
	VAN	Vancomycin	≤ 1	-	≥ 2	CLSI Vet01S, 2020
Protein synthesis inhibitors	AZM	Azithromycin	≤ 0.5	1	≥ 2	CLSI Vet01S, 2020
	CHL	Chloramphenicol	≤ 4	8	≥ 16	CLSI Vet01S, 2020
	CTC	Chlortetracycline	≤ 2	4	≥ 8	Che et al., 2018
	CLI	Clindamycin	≤ 0.5	1-2	≥ 4	CLSI Vet01S, 2020
	ERY	Erythromycin	≤ 0.25	0.5	≥ 1	EUCAST, 2020
	FFC	Florfenicol	≤ 2	4	≥ 8	CLSI Vet01S, 2020
	GEN	Gentamicin	≤ 4	8	≥ 16	Zhang et al., 2020
	LNZ	Linezolid	≤ 2	-	≥ 4	EUCAST 2020
	NEO	Neomycin	≤ 16	-	≥ 32	Gurung et al., 2015
	OXY	Oxytetracycline	≤ 4	-	≥ 8	Gurung et al., 2015
	SPE	Spectinomycin	≤ 64	-	≥ 128	Yu et al, 2018
	TET	Tetracyclin	≤ 0.5	1	≥ 2	CLSI Vet01S, 2020
	TMS	Tiamulin	≤ 16	-	≥ 32	Yu et al., 2018
	TGC	Tigecycline	≤ 0.25	-	≥ 0.5	FDA, 2019
	TMS	Tilmicosin	≤ 16	-	≥ 32	Zhang et al., 2015
	TUL	Tulathromycin	ND	ND	ND	ND
	TYL	Tylosin tartrate	≤ 4	-	≥ 8	Gurung et al, 2015
DNA synthesis inhibitors	ENO	Enrofloxacin	≤ 0.5	1	≥ 2	CLSI Vet01S, 2020
	LEV	Levofloxacin	≤ 0.01	0.03-2	≥ 4	EUCAST, 2020
	MXF	Moxifloxacin	≤ 0.5	-	≥ 1	EUCAST, 2020
Antimetabolites	SDM	Sulphadimethoxine	ND	ND	ND	ND
	SXT	Trimethoprim/sulfamethoxazole	≤ 0.5/9.5	1/19-2/38	≥ 4/76	CLSI Vet01S, 2020

[a] MIC, minimum inhibitory concentration values, are interpreted as susceptible (S), intermediate (I), and resistant (R) using CLSI veterinary breakpoints (Vet01S, 2020), EUCAST (EUCAST, 2020), FDA (FDA, 2019), or previously reported data when available. ND, no data/not determined.

Table S4. Minimum inhibitory concentration (MIC) values distribution, MIC50 and MIC90 values, and resistance rates of 72 *S. suis* strains in 2018.

Antibiotic drugs	MIC breakpoints (μg/mL)			MIC values (μg/mL) [a]																	MIC50	MIC90	S (%)	I (%)	R (%)	MIC ranges
	S	I	R	0.008	0.016	0.03	0.06	0.125	0.25	0.5	1	2	4	8	16	32	64	128	256	512						
Amoxicillin/Clavulanic acid	≤ 8/4	16/8	≥ 32/16									60	2	5	1	4					≤ 2	8	93.1	1.4	5.6	≤ 2 - > 16
Ampicillin	≤ 0.5	1	≥ 2						54	2	1	2	2	2	4	5					≤ 0.25	16	77.8	1.4	20.8	≤ 0.25 - > 16
Cefepime	≤ 2	4	≥ 8							53	7	4	4	2	2						≤ 0.5	4	73.6	ND	26.4	≤ 0.5 - > 8
Cefotaxime	≤ 0.5	-	≥ 1					9	5	14	24	6	5	9							1	> 4	38.9	ND	61.1	≤ 0.12 - > 4
Ceftiofur	≤ 0.5	-	≥ 1						47	9	4	5		3	4						≤ 0.25	2	90.3	0.0	9.7	≤ 0.25 - >8
Ceftriaxone	≤ 0.5	-	≥ 1					9	5	12	28	5	13								1	> 2	36.1	ND	63.9	≤ 0.12 - > 2
Cefuroxime	≤ 0.5	-	≥ 1							23	23	10	6	10							1	> 4	31.9	ND	68.1	≤ 0.5 - > 4
Daptomycin	≤ 1	-	≥ 2				3	23	38	3	2	1	2								0.25	0.5	95.8	ND	4.2	≤ 0.06 - > 2
Ertapenem	≤ 0.5	-	≥ 1							70	2										≤ 0.5	≤ 0.5	97.2	ND	2.8	≤ 0.5 - 1
Meropenem	≤ 2	-	≥ 4						70	2											≤ 0.25	≤ 0.25	100.0	ND	0.0	≤ 0.25 - 0.5
Penicillin	≤ 0.25	0.5	≥ 1			6	1	10	11	10	8	4	7	4	11						0.5	> 8	38.9	13.9	47.2	≤ 0.03 - > 8
Vancomycin	≤ 1	-	≥ 2							70	1			1							≤ 0.5	≤ 0.5	98.6	ND	1.4	≤ 0.5 - > 4
Azithromycin	≤ 0.5	1	≥ 2						2				70								> 2	> 2	2.8	0.0	97.2	≤ 0.25 - > 2
Chloramphenicol	≤ 4	8	≥ 16										16	28	11	10	7				8	32	22.2	38.9	38.9	4 - > 32
Chlortetracycline	≤ 2	4	≥ 8							1	1		2	8	60						> 8	> 8	2.8	2.8	94.4	≤ 0.5 - > 8
Clindamycin	≤ 0.5	1-2	≥ 4						1						1	70					> 16	> 16	1.4	0.0	98.6	0.25 - > 16
Erythromycin	≤ 0.25	0.5	≥ 1						2			2	68								> 2	> 2	2.8	0.0	97.2	≤ 0.25 - > 2
Florfenicol	≤ 2	4	≥ 8								2	18	20	6	26						4	> 8	27.8	27.8	44.4	1 - > 8
Gentamicin	≤ 4	8	≥ 16								8	13	14	3	1	33					8	> 16	48.6	4.2	47.2	≤ 1 - > 16
Linezolid	≤ 2	-	≥ 4							7	35	13	15	2							1	4	76.4	ND	23.6	0.5 - > 4
Neomycin	≤ 16	-	≥ 32										6	22	10	17	17				16	> 32	52.8	ND	47.2	≤ 4 - > 32
Oxytetracycline	≤ 4	-	≥ 8							1	1			3	67						> 8	> 8	2.8	ND	97.2	0.5 - > 8
Spectinomycin	≤ 64	-	≥ 128											4	15	17	1	35			32	> 64	51.4	ND	48.6	≤ 8 - > 64
Tetracycline	≤ 0.5	1	≥ 2									1		1	70						> 8	> 8	0.0	0.0	100.0	2 - > 8
Tiamulin	≤ 16	-	≥ 32							2	2	8	1	3	3	2	51				> 32	> 32	26.4	ND	73.6	≤ 0.5 - >32
Tigecycline	≤ 0.25	-	≥ 0.5			1	26	25	20												0.12	> 0.12	ND	ND	ND	0.03 - > 0.12
Tilmicosin	≤ 16	-	≥ 32										1	1				70			> 64	> 64	2.8	ND	97.2	≤ 4 - > 32
Tulathromycin	ND	ND	ND								1		1		1	1	1	67			> 64	> 64	ND	ND	ND	≤ 1 - > 64
Tylosin tartrate	≤ 4	-	≥ 8								2						70				> 32	> 32	2.8	ND	97.2	1 - > 64
Danofloxacin	ND	ND	ND					2	2	9	21	38									> 1	> 1	ND	ND	ND	≤ 0.12 - > 2
Enrofloxacin	≤ 0.5	1	≥ 2						6	17	7	11	31								2	> 2	31.9	9.7	58.3	0.25 - > 2
Levofloxacin	≤ 0.01	0.03 - 2	≥ 4							24	12	3	5	28							1	> 4	ND	54.2	45.8	≤ 0.5 - > 4
Moxifloxacin	≤ 0.5	-	≥ 1								61	10	1								≤ 1	2	ND	ND	ND	≤ 1 - 4
Sulphadimethoxine	ND	ND	ND																5	67	> 256	> 256	ND	ND	ND	≤ 256 - > 256
Trimethoprim/sulfamethoxazole	≤ 0.5/9.5	1/19 - 2/38	≥ 4/76							26	2	1	5	38							> 2	> 4	36.1	4.2	59.7	≤ 0.5 - > 4

[a] White cells indicate the dilution range tested. Green and red vertical lines respectively describe the susceptible and resistant clinical breakpoints recommended by the CLSI (Vet01S, 2020), EUCAST (EUCAST, 2020), FDA (FDA, 2019), and previously reported data. MIC, minimum inhibitory concentration values, which are interpreted as susceptible (S), intermediate (I), and resistant (R). MIC50, the MIC which inhibits 50% of the isolates tested; MIC90, the MIC which inhibits 90% of the isolates tested; ND, no data/not determined.

Table S5. Minimum inhibitory concentration (MIC) values distribution, MIC_{50} and MIC_{90} values, and resistance rates of 97 *S. suis* strains in 2019.

Antibiotic drugs	MIC breakpoints (μg/mL)			MIC values (μg/mL) [a]																	MIC_{50}	MIC_{90}	S (%)	I (%)	R (%)	MIC ranges
	S	I	R	0.008	0.016	0.03	0.06	0.125	0.25	0.5	1	2	4	8	16	32	64	128	256	512						
Amoxicillin/Clavulanic acid	≤ 8/4	16/8	≥ 32/16									79	4	11	3						≤ 2	8	96.9	3.1	0.0	≤ 2 - 16
Ampicillin	≤ 0.5	1	≥ 2						56	5	1	8	6	4	4	13					≤ 0.25	> 16	62.9	1.0	36.1	≤ 0.25 - > 16
Cefepime	≤ 2	4	≥ 8							69	10	5	9	3	1						≤ 0.5	4	71.1	ND	28.9	≤ 0.5 - > 8
Cefotaxime	≤ 0.5	-	≥ 1					12	10	19	26	9	6	15							1	> 4	42.3	ND	57.7	≤ 0.12 - > 4
Ceftiofur	≤ 0.5	-	≥ 1						55	11	13	4	3	4	7						≤ 0.25	8	85.6	3.1	11.3	≤ 0.25 - > 8
Ceftriaxone	≤ 0.5	-	≥ 1					13	7	20	23	11	23								1	> 2	41.2	ND	58.8	≤ 0.12 - > 2
Cefuroxime	≤ 0.5	-	≥ 1							37	31	13		16							1	> 4	38.1	ND	61.9	≤ 0.5 - > 4
Daptomycin	≤ 1	-	≥ 2				12	41	37	3	2		2								0.12	0	97.9	ND	2.1	≤ 0.06 - > 2
Ertapenem	≤ 0.5	-	≥ 1							95		2									≤ 0.5	≤ 0.5	97.9	ND	2.1	≤ 0.5 - 2
Meropenem	≤ 2	-	≥ 4						96	1											≤ 0.25	≤ 0.25	100.0	ND	0.0	≤ 0.25 - 0.5
Penicillin	≤ 0.25	0.5	≥ 1			5	9	2	8	23	9	4	8	7	22						1	> 8	24.7	23.7	51.5	≤ 0.03 - > 8
Vancomycin	≤ 1	-	≥ 2							93		2	1	1							≤ 0.5	≤ 0.5	95.9	ND	4.1	≤ 0.5 - > 4
Azithromycin	≤ 0.5	1	≥ 2						2	1	1	1	92								> 2	> 2	3.1	1.0	95.9	≤ 0.25 - > 2
Chloramphenicol	≤ 4	8	≥ 16									4	30	21	16	15	11				8	> 32	35.1	21.6	43.3	2 - > 32
Chlortetracycline	≤ 2	4	≥ 8							2	1		1	4	89						> 8	> 8	3.1	1.0	95.9	≤ 0.5 - > 8
Clindamycin	≤ 0.5	1-2	≥ 4										1	2	1	93					> 16	> 16	0.0	0.0	100.0	4 - > 16
Erythromycin	≤ 0.25	0.5	≥ 1						2	1	1	1	92								> 2	> 2	2.1	1.0	96.9	≤ 0.25 - > 2
Florfenicol	≤ 2	4	≥ 8								2	27	18	3	47						8	> 8	29.9	18.6	51.5	1 - > 8
Gentamicin	≤ 4	8	≥ 16								11	15	12	7	10	42					16	> 16	39.2	7.2	53.6	≤ 1 - > 16
Linezolid	≤ 2	-	≥ 4						2	13	31	26	20	5							2	4	74.2	ND	25.8	≤ 0.25 - > 4
Neomycin	≤ 16	-	≥ 32										10	35	18	9	25				16	> 32	64.9	ND	35.1	≤ 4 - > 32
Oxytetracycline	≤ 4	-	≥ 8							1	1	3		1	91						> 8	> 8	5.2	ND	94.8	≤ 0.5 - > 8
Spectinomycin	≤ 64	-	≥ 128											10	17	15	3	52			> 64	> 64	46.4	ND	53.6	≤ 8 - > 64
Tetracycline	≤ 0.5	1	≥ 2								2				95						> 8	> 8	0.0	2.1	97.9	≤ 1 - > 8
Tiamulin	≤ 16	-	≥ 32							4	3	3		2	6	3	76				> 32	> 32	18.6	ND	81.4	≤ 0.5 - > 32
Tigecycline	≤ 0.25	-	≥ 0.5			4	11	14	68												> 0.12	> 0.12	ND	ND	ND	0.03 - > 0.12
Tilmicosin	≤ 16	-	≥ 32											1				96			> 64	> 64	1.0	ND	99.0	8 - > 64
Tulathromycin	ND	ND	ND									1	1		1	1	2	91			> 64	> 64	ND	ND	ND	2 - > 64
Tylosin tartrate	≤ 4	-	≥ 8								2						95				> 32	> 32	2.1	ND	97.9	1 - > 32
Danofloxacin	ND	ND	ND						5	22	20	50									> 1	> 1	ND	ND	ND	0.25 - > 1
Enrofloxacin	≤ 0.5	1	≥ 2						8	30	9		50								> 2	> 2	39.2	9.3	51.5	0.25 - > 2
Levofloxacin	≤ 0.01	0.03 - 2	≥ 4							39	6	2	5	45							4	> 4	0.0	48.5	51.5	≤ 0.5 - > 4
Moxifloxacin	≤ 0.5	-	≥ 1								82	10	4	1							≤ 1	2	ND	ND	ND	≤ 1 - 8
Sulphadimethoxine	ND	ND	ND																5	92	> 256	> 256	ND	ND	ND	≤ 256 - > 256
Trimethoprim/sulfamethoxazole	≤ 0.5/9.5	1/19 - 2/38	≥ 4/76							35	1	2	10	49							> 2	> 4	36.1	3.1	60.8	≤ 0.5 - > 4

[a] White cells indicate the dilution range tested. Green and red vertical lines respectively describe the susceptible and resistant clinical breakpoints recommended by the CLSI (Vet01S, 2020), EUCAST (EUCAST, 2020), FDA (FDA, 2019), and previously reported data. MIC, minimum inhibitory concentration values, which are interpreted as susceptible (S), intermediate (I), and resistant (R). MIC_{50}, the MIC which inhibits 50% of the isolates tested; MIC_{90}, the MIC which inhibits 90% of the isolates tested; ND, no data/not determined.

Table S6. Minimum inhibitory concentration (MIC) values distribution, MIC_{50} and MIC_{90} values, and resistance rates of 77 *S. suis* strains in 2020.

Antibiotic drugs	MIC breakpoints (μg/mL)			MIC values (μg/mL)[a]																	MIC_{50}	MIC_{90}	S (%)	I (%)	R (%)	MIC ranges
	S	I	R	0.008	0.016	0.03	0.06	0.125	0.25	0.5	1	2	4	8	16	32	64	128	256	512						
Amoxicillin/Clavulanic acid	≤ 8/4	16/8	≥ 32/16									55	7	11	4						≤ 2	8	94.8	5.2	0.0	≤ 2 - 16
Ampicillin	≤ 0.5	1	≥ 2						40	4	4	5	5	1	5	13					≤ 0.25	> 16	57.1	5.2	37.7	≤ 0.25 - > 16
Cefepime	≤ 2	4	≥ 8							46	9	9	5	5	3						≤ 0.5	4	59.7	ND	40.3	≤ 0.5 - > 8
Cefotaxime	≤ 0.5	-	≥ 1					6	5	19	14	13	1	19							1	> 4	39.0	ND	61.0	≤ 0.12 - > 4
Ceftiofur	≤ 0.5	-	≥ 1						40	4	12	6	2	8	5						≤ 0.25	8	80.5	2.6	16.9	≤ 0.25 - > 8
Ceftriaxone	≤ 0.5	-	≥ 1					8	4	14	17	6	28								1	> 2	33.8	ND	66.2	≤ 0.12 - > 2
Cefuroxime	≤ 0.5	-	≥ 1							19	22	15	1	20							1	> 4	24.7	ND	75.3	≤ 0.5 - > 4
Daptomycin	≤ 1	-	≥ 2				7	34	32		2		2								0.12	0.25	97.4	ND	2.6	≤ 0.06 - > 2
Ertapenem	≤ 0.5	-	≥ 1							75	1		1								≤ 0.5	≤ 0.5	97.4	ND	2.6	≤ 0.5 - 4
Meropenem	≤ 2	-	≥ 4						75	1	1										≤ 0.25	≤ 0.25	100.0	ND	0.0	≤ 0.25 - 1
Penicillin	≤ 0.25	0.5	≥ 1			4		8	9	8	2	8	12	5	21						2	> 8	27.3	10.4	62.3	≤ 0.03 - > 8
Vancomycin	≤ 1	-	≥ 2							74	1	1		1							≤ 0.5	≤ 0.5	97.4	ND	2.6	≤ 0.5 - > 4
Azithromycin	≤ 0.5	1	≥ 2						3			1	73								> 2	> 2	3.9	0.0	96.1	≤ 0.25 - > 2
Chloramphenicol	≤ 4	8	≥ 16									1	18	23	14	13	8				8	32	24.7	29.9	45.5	2 - > 32
Chlortetracycline	≤ 2	4	≥ 8							1	1			5	70						> 8	> 8	2.6	0.0	97.4	≤ 0.5 - > 8
Clindamycin	≤ 0.5	1-2	≥ 4										1		1	75					> 16	> 16	0.0	0.0	100.0	4 - > 16
Erythromycin	≤ 0.25	0.5	≥ 1						2		2	3	70								> 2	> 2	2.6	0.0	97.4	≤ 0.25 - > 2
Florfenicol	≤ 2	4	≥ 8								2	13	20	5	37						8	> 8	19.5	26.0	54.5	1 - > 8
Gentamicin	≤ 4	8	≥ 16								5	7	21	6		38					8	> 16	42.9	7.8	49.4	≤ 1 - > 16
Linezolid	≤ 2	-	≥ 4							5	32	17	17	6							2	4	70.1	ND	29.9	0.5 - > 4
Neomycin	≤ 16	-	≥ 32										10	13	23	7	24				16	> 32	59.7	ND	40.3	≤ 4 - > 32
Oxytetracycline	≤ 4	-	≥ 8							1		1		2	73						> 8	> 8	2.6	ND	97.4	≤ 0.5> 8
Spectinomycin	≤ 64	-	≥ 128											3	14	8	3	49			> 64	> 64	36.4	ND	63.6	≤ 8 - > 64
Tetracycline	≤ 0.5	1	≥ 2									2		1	74						> 8	> 8	0.0	0.0	100.0	2 - > 8
Tiamulin	≤ 16	-	≥ 32							3	3	1	1	2	4	2	61				> 32	> 32	18.2	ND	81.8	≤ 0.5> 32
Tigecycline	≤ 0.25	-	≥ 0.5		2	14	16	29	16												0.12	> 0.12	ND	ND	ND	≤ 0.015 - > 0.12
Tilmicosin	≤ 16	-	≥ 32										2			1		74			> 64	> 64	2.6	ND	97.4	≤ 4 - > 64
Tulathromycin	ND	ND	ND									2					1	74			> 64	> 64	ND	ND	ND	2 - > 64
Tylosin tartrate	≤ 4	-	≥ 8									1				1	75				> 32	> 32	1.3	ND	98.7	2 - > 32
Danofloxacin	ND	ND	ND						1	20	12	44									> 1	> 1	ND	ND	ND	0.25 - > 1
Enrofloxacin	≤ 0.5	1	≥ 2					1	6	23	4	1	42								> 2	> 2	39.0	5.2	55.8	≤ 0.12 - > 2
Levofloxacin	≤ 0.01	0.03 - 2	≥ 4							20	14	3	2	38							4	> 4	ND	48.1	51.9	≤ 0.5 - > 4
Moxifloxacin	≤ 0.5	-	≥ 1								63	7	7								≤ 1	2	ND	ND	ND	≤ 1 - 4
Sulphadimethoxine	ND	ND	ND																3	74	> 256	> 256	ND	ND	ND	≤ 25 - 6> 256
Trimethoprim/sulfamethoxazole	≤ 0.5/9.5	1/19 - 2/38	≥ 4/76							19	2		11	45							> 4	> 4	24.7	2.6	72.7	≤ 0.5 - > 4

[a] White cells indicate the dilution range tested. Green and red vertical lines respectively describe the susceptible and resistant clinical breakpoints recommended by the CLSI (Vet01S, 2020), EUCAST (EUCAST, 2020), FDA (FDA, 2019), and previously reported data. MIC, minimum inhibitory concentration values, which are interpreted as susceptible (S), intermediate (I), and resistant (R). MIC_{50}, the MIC which inhibits 50% of the isolates tested; MIC_{90}, the MIC which inhibits 90% of the isolates tested; ND, no data/not determined.

Table S7. Oligonucleotide primer sequences (Kerdsin et al., 2014).

Multiplex PCR reaction set	Target genes or serotypes	Nucleotide sequences (5′–3′)	Amplicon size (bp)
All	*gdh*	F: TTCTGCAGCGTATTCTGTCAAACG	695
		R: TGTTCCATGGACAGATAAAGATGG	
1	1 or 14	F: AATCATGGAATAAAGCGGAGTACAG	550
		B: ACAATTGATACGTCAAAATCCTCACC	
	2 or ½	F: GATTTGTCGGGAGGGTTACTTG	450
		B: TAAATAATATGCCACTGTAGCGTCTC	
	3	F: TGGGAGAAGGCAGAAAGTACGAGA	1,273
		B: ACCCCCAGAAGAGCCGAAGGA	
	7	F: GATGATTTATGGCACCCGAGTAAGC	150
		B: AGTCACAATTGCTGGTCCTGACACC	
	9	F: GGGATGATTGCTCGACAGAT	300
		B: CCGAAGTATCTGGGCTACTG	
	11	F: TACAGTGCTTGCAGCCCTAC	896
		B: CGACTTGTCGTGCCCTGAT	
	16	F: TGGAGGAGCATCTACAGCTCGGAAT	202
		B: TTTGTTTGCTGGAATCTCAGGCACC	
2	4	F: ACTTGGAGTTGTCGGAGTAGTGCT	783
		B: ACCGCGATGGATAGGCCGAC	
	5	F: TGATGGCGGAGTTTGGGTCGC	166
		B: CGTAACAACCGCCCCAGCCG	
	8	F: ATGGGCGTTGGCGGGAGTTT	320
		B: TTACGGCCCCCATCACGCTG	
	12	F: TGTGGCGATAGGACAACAGG	209
		B: ACCAAGAAGTTTCCGCCTGA	
	18	F: CGGGGCAGTCTTACTCATGG	432
		B: ATGACAGCGAAACGGACAGA	
	19	F: AGCAGGGTTGCGTATGGCGG	1,024
		B: ACAAGCACCAGCAAAGACCGCA	
	24	F: ACCCGGAAAAACCAGGAGTT	500
		B: ACCAATCAATGCCAAGCGAC	
	25	F: GGAGGAGCTGCGGGCTCATA	1211
		B: TGGCCACAACCTGGATGCGTT	
3	6	F: TACGGTCTCCCTTGCCTGTA	325
		B: AACTCAGCTAGTGCTCCACG	
	10	F: TTACGAGGGGATTCTGGGGT	153
		B: CGGGACAACAGATGGAACCT	
	13	F: CTGGTGCTGCAATTTCGCTT	1,135
		B: GCAGACTAGCTGCAGTTCCA	
	15	F: GCAAGAAAGCTTCCGGATGGA	274
		B: CAAGAGAGTGTGCAACCCCA	
	17	F: ACTTGGGTTGGAATGGCGAA	906
		B: ACCACCGAAAGTCAGGTCAC	
	23	F: TGCTCAACAAACGCAGCAAA	454
		B: TGACTGGTACATCTGCAGCC	
	31	F: GGAGTGCTCTATGCCACCTT	550
		B: GCATTGCCCCTACAGCAAAC	
4	21	F: GGTGGCAAGGAGAGCAAAGT	325
		B: ACATGGTAAGCCATTGCTGGA	
	27	F: CTACGCCAATCGAAGCCAGA	506
		B: CCAGTAAGAAGCCTGTCGCA	
	28	F: GGACTTCGGTACCTTAGCGT	865
		B: CTCCAGCACATTCCCGTACC	
	29	F: GTGCGGGCGTTATTTTTGGT	435
		B: AGCCTTGCAACCCATTTCCT	
